# Determinants of childhood diarrhea in West Gojjam, Northwest Ethiopia: a case control study

**DOI:** 10.11604/pamj.2018.30.234.14109

**Published:** 2018-07-27

**Authors:** Meskerem Girma, Tesfaye Gobena, Girmay Medhin, Janvier Gasana, Kedir Teji Roba

**Affiliations:** 1 Ethiopian Institute of Water Resources, Addis Ababa University, Addis Ababa, Ethiopia; 2 College of Health and Medical Science, Haramaya University, Dire Dawa, Ethiopia; 3 Aklilu Lemma Institute of Pathobiology, Addis Ababa University, Addis Ababa, Ethiopia; 4 Kuwait University Health Sciences Center Faculty of Public Health, Kuwait; 5 College of Health and Medical Science, Haramaya University, Dire Dawa, Ethiopia

**Keywords:** Childhood diarrhea, hygiene, water, determinant, case control, Ethiopia

## Abstract

**Introduction:**

Childhood diarrhea is a global public health problem that affects both developed and developing countries including Ethiopia. The objective of this study was to assess determinants of childhood diarrhea among children under-five years of age in West Gojjam Zone, northwest Ethiopia.

**Methods:**

A community-based case control study was conducted in four districts of West Gojjam in the northwest of Ethiopia from July to August, 2015. A randomly selected sample of 118 cases and 351 controls who met the inclusion criteria were included in this study. Data were collected using a structured questionnaire through face to face interview. Independent variables which had p-value less than 0.2 at an unadjusted model were candidate for the final model. Adjusted odds ratio was used to control confounding effects and to determine predictors of an outcome.

**Results:**

Unimproved water sources (AOR, 1.88; 95 % CI: 1.17-3.03), lack of hand washing at critical times (AOR, 2.38; 95 % CI: 1.42-3.99) and a deepening method to take water from a water storage container (AOR, 2.11; 95 % CI: 1.28-3.47), presence of two or more young siblings (AOR, 4.15; 95 % CI: 2.57-6.70), rural residence (AOR,2.11 95 % CI: 2.21-3.68), and not using latrine for disposal of child feces (AOR, 1.90; 95 % CI: 1.12-3.22) were predictors of diarrhea among children under the age of five.

**Conclusion:**

The majority of the causes of childhood diarrhea in the study area were preventable. Thus, health extension workers should give tailored health information to mothers or caregivers on the importance of sanitation, personal and environmental hygiene and drinking water handling methods.

## Introduction

Childhood diarrhea remains a global problem associated with high mortality and morbidity rates affecting both developed and developing countries [1, 2]. The prevalence of diarrhea in the population is known to differ among different groups, but children under the age of five years are among the most affected social groups [3]. A wide range of pathogens can cause diarrhea including bacteria, viruses and protozoa and is often transmitted to humans through contaminated food or water [4, 5]. According to World Health Organization's (WHO) estimates, globally 1.7 billion cases of diarrhea among children under-five years of age occur each year, one of the leading infectious diseases causing deaths worldwide and the second most prevalent disease responsible for 578,000 deaths in 2013 [6]. The greatest proportions of severe episodes of diarrhea occurred in the Southeast Asian (26 %) and African regions (26 %) in 2010 [7]. Even if mortality from diarrhea has declined considerably over the past years globally, morbidity from diarrhea in Africa has not; in sub-Saharan Africa, diarrhea accounted for 25 to 75% of childhood morbidity and 50% of childhood mortality [7]. Determinants contributing to the occurrence of childhood diarrhea include low maternal education [8, 9], number of children under the age of five [10], availability of latrine [11], poor handling of drinking water [12, 13], and weather events [14]. Systematic reviews indicated that diarrhea is highly prevalent in areas with unimproved drinking water supply, poor hygiene, and unavailability of toilets [15, 16]. In Ethiopia, provision of water supply and improvement in sanitation and hygiene have shown progress in the past ten years. According to WHO, availability of improved drinking water supply in Ethiopia increased from 13% in 1990 to 57% in 2015 [17]. The availability of toilets increased gradually in the Amhara Region from 2% in 2000 to 46% in 2012 [18]. However, based on community-based research and facility-based reports we can conclude that childhood diarrhea continues to be one of the top five leading causes of morbidity and mortality in developing countries over the past decades, including West Gojjam, northwest Ethiopia [7, 19]. For effective planning and implementation of prevention strategies, a situational analysis of the determinants of childhood diarrhea is needed. The objective of this study was to assess the determinants of childhood diarrhea among children under-five years of age in West Gojjam, northwest Ethiopia.

## Methods

**Figure 1:**
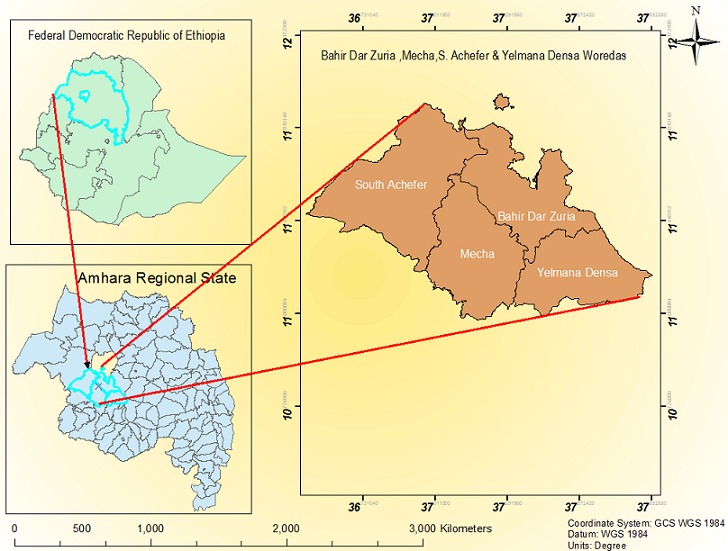
Map showing West Gojjam and the location of the four different districts (*woredas*)

**Study area:** This study was conducted in rural *kebeles* (neighborhoods or the smallest administrative units of Ethiopia) of four districts of West Gojjam Zone of the Amhara Regional State (Figure 1). Based on the 2007 population and housing census [20], the population of West Gojjam Zone was 2,106,596 of which 1,058,272 are male and 1,048,324 are female. The zone has an area of 13,311.94 square kilometers. The dominant ethnic group of the zone is Amhara, and agriculture is the main livelihood of the population with *teff*, maize, millet, barley and legumes being the main crops cultivated in the districts of the zone.

**Study design and period:** Case control study designed was used and the research was conducted in four randomly selected districts (Debub Achefer, Bahir Dar Zuria, Mecha and Yilmana Densa) from July to August, 2015.

**Sample size determination:** The sample size was determined using Epi Info Version 3.5.2 statistical software by considering the following assumptions: 95% significance level, 80% power, P1= proportion of diarrheic children with unimproved source of water, P2 = proportion of children non-diarrheic with unimproved source of water as main predictors of the outcome (diarrhea) from studies conducted in the Derashe District, which was 53.26% for cases and 37.15% for controls [11]. The proportion of case and control was assumed to be 1:3. Therefore P1=0.5326 Zα/ 2=1.96 (95% CI) and P2=0.3715, Zβ =0.84 (power of 80). By considering a10 % non-response rate, the final sample size was 469 (118 cases and 351 controls).

**Sampling techniques:** A multistage sampling technique was used to select the participants of the study. First, four districts were selected randomly from thirty districts. All children under-five years of age living in randomly selected districts were the population of the study. Second, a total of 16 kebeles (one urban and three rural kebeles from each of the four randomly selected districts) were chosen using simple random sampling technique. Then, all households with children under- five years of age in the selected kebeles were registered via a house-to-house survey, in order to register all under-five children with or without diarrhea. Living in the study area for at least 6 months was used as a selection criterion. After identifying cases and controls, the study participants were selected using simple random sampling technique.

**Measurement:** Explanatory variables include sociodemographic characteristic such as age of children, educational and occupational status of mothers or caregivers, family size and housing condition. Waste disposal and water supply factors include waste disposal, water sources, availability and utilization of latrine. Childcare related behavior factors include hand washing at critical times, drinking water handling, breast feeding status and supplementary feeding.

**Operational definitions:** Diarrhea among under-five children was diagnosed using the WHO recommendation that identifies a child with diarrhea as having three or more loose stools or watery diarrhea in a day during the two weeks preceding the study [21]. Case refers to participating children who had diarrhea during the two weeks prior to the survey. Control denotes participating children free of diarrhea during the two weeks prior to the survey. Hand washing at critical times includes hand washing after visiting a toilet, before eating food, after cleaning a child's bottom, before preparing food and after feeding children.

**Data collection procedure:** A questionnaire was developed by reviewing the related literature. Data were collected through face-to-face interviews of mothers or caregivers of the children using a pretested questionnaire. In addition, an observational checklist was used to observe water storage containers, presence or absence of kitchens, availability and types of latrine, and presence or absence of hand washing facilities. The data were collected by female nurses with a qualification of a diploma who could speak the local language (Amharic) and who were aware of the norms of the society. Data collectors with previous data collection experiences were recruited and trained for five days in how to administer the instruments. Four supervisors and a principal investigator of the study checked the collected data for completeness, and consistency on a daily basis.

**Quality assurance:** A structured pretested questionnaire was used for data collection. The questionnaire was initially prepared in English and translated into Amharic, which is a local language, and it was again translated back into English to ensure its consistency. Corrections and modifications were made as deemed necessary based on comments and suggestions obtained from the pretest. Data collectors who had the experience of working in similar studies in the past were selected, and they were given training to hone their skills. The supervisor and the principal investigator checked the collected data for completeness, clarity and consistency on a daily basis.

**Data processing and analysis:** Before data entry, the questionnaires were checked for completeness and consistency. Then, the data were coded and double entered into Epi Info Version 3.5.4 and were exported to SPSS Version 20 for data processing and analysis. Descriptive statistical analyses were computed to calculate means, standard deviations, frequencies and percentages. Logistic regression analysis was performed separately for three variable blocks to estimate the effects of socio-economic, environmental, and childcare related behavior factors. Crude odds ratios and corresponding 95% confidence intervals were used to quantify an unadjusted strength of association between the independent and the outcome variables. Independent variables which resulted in a p-value less than 0.2 in an unadjusted model were candidates to be considered for the final multivariable model. Multivariable logistic regression was fitted to obtain adjusted odds ratios after controlling the confounding effects of different variables and to determine factors associated with the outcome variable. All variables included in the final model and resulted in a p-value less than 5% had statistically significant association with the outcome.

**Ethics approval and consent to participate:** The ethical clearance was obtained from Institutional Ethical Committee (IEC) of Ethiopian Institute of Water Resources, Addis Ababa University. Permission was obtained from West Gojjam Administration and also from health offices in each district and from each respective kebele administration. The purpose of the study was explained to the participants, and written consent was obtained from mothers or caretakers participating in the study. Confidentiality was maintained during the interview process by avoiding mentioning names and other personal identification information. Moreover, information about the purpose of the study, the procedures, the risks and benefits of this research was provided for all participants.

## Results

**Sociodemographic characteristics of study participants:** A total of 118 cases and 351 controls were involved in this study. The response rate for the study was 100%. The mean age of mothers or caregivers was 28.2 (SD ± 5.6) years with a range of 18-57 years. The majority of the mothers were found in the age range of 25-34 years. The mean age of the children was 24.8 (SD ± 9.8) months, and more than half of the children (56.7%) were males. More than one-third of the children were found in the age range of 24-59 months and most participants (79.7%) were living in rural areas. About 67.4% of the mothers had no formal education (Table 1).

**Table 1:** Sociodemographic, waste disposal and water supply factors in West Gojjam, northwest Ethiopia, July, 2015

Variables	Diarrhea status		COR(95%CI)	P-value
Age of child	Cases (%)	Controls (%)		
0-5	12(10.3)	41(11.7)	1.00	
6-11	43(36.4)	75(21.4)	0.51(0.24-1.07)	0.49
12-23	22(18.6)	99(28.2)	1.32(0.59-2.91)	0.77
24-59	41(34.7)	136(38.7)	0.97(0.47-2.02)	0.93
**Child gender**				
Male	68(57.6)	198(56.4)	0.95(0.62-1.45)	0.82
Female	50(42.4)	153(43.6)	1.00	
**Number of under-five children**				
One	46(39.0%)	252(71.8%)	1.00	<0.01
Two and above	72(61.0%)	117(33.3%)	3.98(2.57-6.16)	
**Education mother/caretaker**				
No formal education	89(75.4%)	227(64.7%)	1.676(1.04-2.69)	0.03
Formal education	29(24.6%)	124(35.3%)	1.00	
**Residence**				
Rural	94(79.7)	234(66.3)	1.96(1.19-3.23)	0.01
Urban	24(20.3)	117(33.7)	1.00	
**Water source**				
Improved	56(47.5)	233(66.4)	1.00	
Unimproved	62(52.5)	118(33.6)	2.19(1.43-3.34)	**<**0.01
**Latrine availability**				
Yes	77(65.3)	282(80.3)	1.00	
No	41(34.7)	69(19.7)	2.17(1.37-3.65)	0.03
**Refuse Disposal**				
Proper disposal method	73(61.9)	242(68.9)	1.00	
Improper disposal method	45(38.1)	109(31.1)	1.37(0.88-2.11)	0.16
**Adult member defecation**				
Use open field/river basin	38(32.2)	62(17.7)	2.21(1.38-3.55)	**<**0.01
Use latrine	80(67.8)	289(82.3)	1.00	

Odds ratio (OR), confidence interval (CI) and P-value derived from bivariate logistic regression based on likelihood ratio test, significant P-value of the models are indicated in bold letter.

**Determinants of childhood diarrhea:** The majority (61.9%) of the households among cases and 68.9% of the controls used proper methods of waste disposal. Having a latrine in the households was 65.3% among cases and 80.3% among controls. About 47.5% from cases and 66.4% from controls of households in the four districts used improved water source. In addition, more than 29.7% of cases and 47.6% controls in the districts used pouring method to take water from storage container (Table 1). The proportion of children vaccinated against measles was 86.4% among cases and 88.9% among controls. Among controls, 41.9% of the mothers or caregivers reported that they washed their hands at least at three critical hand washing times compared to 30.5% among cases. Almost 71% of the controls breastfed their children for more than one year compared with only 61.9% of cases. The majority of the households (80.9%) among controls and 60.2% among cases were using latrines for disposal of child feces (Table 2). In the bivariate model, educational status of mothers or caregivers, latrine availability, adult member defecation practice, and hand washing practice at critical times were significantly associated with diarrhea. After controlling potential confounding variables using multivariate analysis the following variables were significant predictors of diarrhea among children of under-five years: number of under- five children in a household, residence, water source, disposal of child feces, separate feeding material and method of drawing drinking water from a water storage container. Childhood diarrhea was significantly higher if households were users of unimproved water source (AOR 1.9; 95% CI 1.2-3.0), having two or more siblings compared to children in the households with only one sibling (AOR 4.1; 95% CI 2.6-6.7), not using a latrine for disposal of child feces (AOR 1.9; 95% CI 1.1-3.2), lack of practice of hand washing at critical times (AOR 2.4; 95% CI 1.4-3.9), living in a rural area (AOR ; 2.1 95% CI 2.2-3.7), and practicing a deepening method to take water from a water storage container (AOR 2.1; 95% CI 1.3-3.5) (Table 3).

**Table 2:** Maternal child caring behavior factors and childhood diarrhea in West Gojjam, northwest Ethiopia, July, 2015

Variables	Diarrhea status		COR(95%CI)	P-value
	Cases (%)	Controls (%)		
**Method of drawing**				
Deepening	83(70.3)	184(52.4)	2.15(1.38-3.37)	<0.01
Pouring	35(29.7)	167(47.6)	1.00	
**Hand washing at critical times**				
Yes	36(30.5)	147(41.9)	1.00	
No	82(69.5)	204(58.1)	1.64(1.052.56)	0.03
**Separate feeding materials**				
Yes	48(40.7)	252(71.8)	1.00	
No	70(59.3)	99(28.2)	3.71(1.13-2.70)	0.01
**Child Feces disposal**				
latrine	71(60.2)	284(80.9)	2.81(1.78- 4.42)	<0.01
open field	47(39.8)	67(19.1)	1.00	
**Measles**				
Yes	102(86.4)	312(88.9)	1.00	
No	16(13.6)	39(11.1)	1.12(0.59-2.12)	0.73
**Duration of breast feeding**				
< 1 year	47(39.8)	99(28.2)	1.00	
> 1 year	71(60.2)	252(71.8)	1.68(1.09-2.60)	0.02

ratio (OR), confidence interval (CI) and P-value derived from bivariate logistic regression based on likelihood ratio test, significant P-value of the models are indicated in bold letter

**Table 3:** Variables significantly associated with childhood diarrhea in West Gojjam, northwest Ethiopia, July, 2015

Variables	Model 2 AOR(95%CI)	Model 3 AOR(95%CI)	Model 4 AOR(95%CI)
**Number of under-five children**			
One	1.00		1.00
Two and above	4.04(2.56-6.39)		4.15(2.57-6.70)**
**Education of mother/caretaker**			
No formal education	0.63(0.37-1.05)		0.75(0.43-1.30)
Formal education	1.00		1.00
**Residence**			
Rural	2.14(2.26-3.66)		**2.11(2.21-3.68)** *
Urban	1.00		1.00
**Water source**			
Improved	1.00		1.00
Unimproved	2.12(1.33-3.32)		**1.88(1.17-3.03)** *
**Latrine availability**			
Yes	1.00		1.00
No	0.48(0.23-1.03)		0.59(0.27-1.28)
**Adult member defecation**			
Use open field/river basin	1.19(0.54-2.63)		1.47(0.64-3.45)
Use latrine	1.00		1.00
**Method of drawing**			
Deepening		2.30(1.44-3.68)	2.11(1.28-3.47)*
Pouring		1.00	1.00
**Hand washing at critical times**			
Yes		1.00	1.00
No		1.83(1.15-2.92)	2.38(1.42-3.99)**
**Separate feeding Materials**			
Yes		1.00	1.00
No		1.62(1.01-2.59)	1.55(0.91-2.62)
**Child Feces disposal**			
Latrine		1.00	1.00
Open field		2.54(1.58-4.09)	1.90(1.12-3.22)*
**Duration of breast feeding**			
< 1 year		1.77(1.12-2.79)	1.09(0.66-1.79)
> 1 year		1.00	1.00

Odds ratio (OR), confidence interval (CI) and P-value derived from multivariate logistic regression based on likelihood ratio test, significant CI of the models are indicated in bold letter.

* p < 0.05;

** p < 0.001.

## Discussion

This study identified the determinants of diarrhea among children under-five years in West Gojjam Zone, northwest Ethiopia, as unimproved water sources, unavailability of latrine for disposal of child feces, lack of hand washing at critical times and a deepening method to take water from a water storage container, presence of two or more young siblings and being a rural resident. This study indicated that presence of diarrhea was significantly associated with children living in a household with at least two under-five children. This finding supports previous findings from Cameron and Pakistan [22, 23]. This can be justified by the fact that when the number of children in the household increases, the quality of care and attention from parents decreases as mothers or caregivers become incapable of caring for children, so it is expected that children could be more vulnerable to contamination. Furthermore, children who contract a diarrheal disease may easily transmit the disease to others who live in the same household [24]. It is suggested that child spacing services which might have a positive influence on the reduction of the prevalence of diarrhea be provided. Our study showed that being a rural resident was significantly associated with childhood diarrhea. This could be attributed to different factors including lack of basic services to safe water supply, unavailability of latrines and lack of access to solid waste disposal facilities, which are common problems among rural areas than in urban areas [25]. The finding is similar to a study conducted in Uganda which reported a significant association between being a rural resident and having childhood diarrhea [8]. The result of this study regarding water sources indicates that living in a household with unimproved water sources put children at higher risk of having diarrhea, which is in line with a study that recommended the provision of protected water supply as an effective way to reduce the prevalence of childhood diarrhea [26, 27].

This study also revealed that households not using latrines for disposal of child feces tended to increase childhood diarrhea compared to households using latrines for disposal of child feces. A similar study showed that presence of excreta in the yard showed strong association with childhood diarrheal [11]. This is an important finding which showed that the mere presence of a latrine does not ensure the prevention of childhood diarrhea. This study found that the prevalence rate of childhood diarrhea was higher among children in a household whose mothers used a deepening method to take water from a water storage container than among children whose mothers did not use this method. Even though the source is protected, water may be contaminated at or following collection, i.e. during transport, storage and/or by the method to take water from a water storage container. The finding of the study is consistent with previous studies, which found higher probability of childhood diarrhea among children whose mothers failed to wash their hands at critical times [28].

**Strengths and limitations of the study:** One of the strengths of this study was that it was community-based. The other strengths were the fact that it had a high response rate and addressed childhood diarrhea. One of the limitations was the use of case control study design with known drawbacks. The results might have been biased because of potential recall bias; however, this was minimized by using reported-incident cases within a two-week period. Some behavioral practices including hand washing practices used in the analysis were self-reported by the respondents; self-reported data have been found to introduce inaccuracy and bias into estimates of behavior.

**Funding:** The research was funded by the Ethiopian Institute of Water Resources of Addis Ababa University and Haramaya University. The funders had no responsibility in the study design, data collection, data analysis, interpretation of the data, and preparation of the manuscript for publication.

## Conclusion

The determinants of childhood diarrhea in this study were having two and more young siblings, being a rural resident, unimproved water sources, not using latrines for disposal of child feces, lack of hand washing at critical times, and a deepening method to take water from a water storage container. Furthermore, attention should be given to overall hygienic practices of disposing of child feces, and disposal of child feces should get emphasis so as to control and prevent childhood diarrhea. Therefore, health extension workers should give tailored health information to mothers or caregivers on the importance of sanitation, hygiene and childbirth spacing.

### What is known about this topic

- Childhood diarrhea is one of the most common health problems in Ethiopia;
- Improved sanitation and safe water access coverage in Ethiopia are very low.

### What this study adds

- The prevalence of childhood diarrhea is higher among the children whose mothers practice a deepening method to take water from a water storage container. This information can inform local health departments and health workers should give tailored health information to mothers on the importance of proper drinking water handling;
- Most factors that lead to diarrhea among children under-five years of age were easily preventable. This points out the need for promotion and advocacy of hygiene and behavioral intervention measures.

## Competing interests

The authors declare no competing interests.
